# Fermented Ginseng Extract, BST204, Suppresses Tumorigenesis and Migration of Embryonic Carcinoma through Inhibition of Cancer Stem Cell Properties

**DOI:** 10.3390/molecules25143128

**Published:** 2020-07-08

**Authors:** Jong Woo Park, Jee Hun Park, Jeung-Whan Han

**Affiliations:** School of pharmacy, Sungkyunkwan University, Suwon 16419, Korea; jongwoopark17@gmail.com (J.W.P.); croi89@naver.com (J.H.P.)

**Keywords:** cancer stem cell, BST204, ginsenoside, Rh2, Rg3, tumorigenesis, epithelial–mesenchymal transition, invasion, CD133

## Abstract

The pharmacological effects of BST204—a fermented ginseng extract—on several types of cancers have been reported. However, the effects of ginseng products or single ginsenosides against cancer stem cells are still poorly understood. In this study, we identified the anti-tumorigenic and anti-invasive activities of BST204 through the suppression of the cancer stem cell marker, CD133. The treatment of embryonic carcinoma cells with BST204 induced the expression of the tumor suppressor protein, p53, which decreased the expression of cell cycle regulatory proteins and downregulated the expression of CD133 and several stemness transcription factors. These changes resulted in both the inhibition of tumor cell proliferation and tumorigenesis. The knockdown of CD133 suggests that it has a role in tumorigenesis, but not in cancer cell proliferation or cell cycle arrest. Treatment with BST204 resulted in the reduced expression of the mesenchymal marker, N-cadherin, and the increased expression of the epithelial marker, E-cadherin, leading to the suppression of tumor cell migration and invasion. The knockdown of CD133 also exhibited an anti-invasive effect, indicating the role of CD133 in tumor invasion. The single ginsenosides Rg3 and Rh2—major components of BST204—exhibited limited effects against cancer stem cells compared to BST204, suggesting possible synergism among several ginsenoside compounds.

## 1. Introduction

Despite the current advances in cancer biology, and despite the availability of various advanced therapies, cancer remains a leading cause of death globally. The World Health Organization has reported that cancer was responsible for an estimated 9.6 million deaths in 2018, i.e., about one in every six deaths [1]. Cancer recurrence, metastasis, and resistance to various therapies are considered to be the possible obstacles that need to be overcome to combat cancer. Cancer stem cells (CSCs), which are also known as tumor-initiating cells, have been suggested to be responsible for cancer relapse and drug resistance due to their property of self-renewal and capacity to differentiate into heterogeneous lineages of cancer cells [2,3]. Many distinct CSC markers have been identified from different types of tumors, which can be used as possible molecular targets for CSC therapy [4,5]. Among these, CD133 is a well-known marker for a variety of somatic stem cells, early progenitor cells, and CSCs. It can be used as a specific marker for hematopoietic stem cells, neural stem cells, and prostate stem cells. CD133^+^ cells exhibit the potential to self-renew and differentiate [6,7,8]. CD133 can also be used for the identification and characterization of CSCs from brain tumors, as well as from colon, pancreas, and liver cancers [9,10,11,12]. However, CD133 cannot be used as an exclusive selective maker for somatic stem cells or CSCs, but instead needs to be used in combination with other markers. CD133 is a transmembrane glycoprotein localized to membrane protrusions [13]; however, its specific role in CSCs has not been clearly identified. In addition to cell surface markers, several intracellular markers have also been identified. Most importantly, the major stemness-related transcriptional factors, including Nanog, Oct4, and Sox2, have been found to be highly expressed in CSCs [14,15,16]. Many studies have shown a correlation between the expression of CD133 and that of stemness-related transcriptional factors, supporting the proposed role of CD133 in conferring cancer stemness and self-renewal potential in CSCs [17,18,19,20].

Previously, we reported that embryonic carcinoma (EC) cells express high levels of stemness-related transcription factors, and that targeting one of the factors, Nanog, using a histone deacetylase inhibitor can serve as an improved therapeutic strategy for poorly differentiated cancer stem-like cells, involving the suppression of EC proliferation and tumorigenesis [21]. We further showed that CD133 is also highly expressed in EC cells and negatively regulated by the tumor suppressor protein, p53 [20].

The root of ginseng, *Panax ginseng* Meyer (Araliaceae), has been widely used as a herbal medicine in Asian countries. Ginseng contains many different ingredients, including saponins, phenolic compounds, polyacetylenes, alkaloids, polysaccharides, and most importantly ginsenosides, which are the major active compounds with various pharmacological effects, such as anti-inflammatory, anti-carcinogenic, and cardiovascular protective activities [22,23,24,25]. Among all the ginsenosides, Rh2 and Rg3 have shown anti-carcinogenic effects against a variety of cancers [26,27,28].

BST204 is a ginseng extract that is fermented using the enzyme ginsenoside-β-glucosidase, resulting in the enrichment of Rh2 and Rg3. It has diverse biological activities, including anti-inflammatory, anti-carcinogenic, and anti-adipogenic effects [29,30,31]. In this study, we investigated the anti-tumorigenic and anti-invasive activities of BST204 through the suppression of the cancer stem cell marker in EC cells.

## 2. Results

### 2.1. BST204 Inhibits Proliferation of EC Cells Through G1 Cell Cycle Arrest

**Figure 1:**
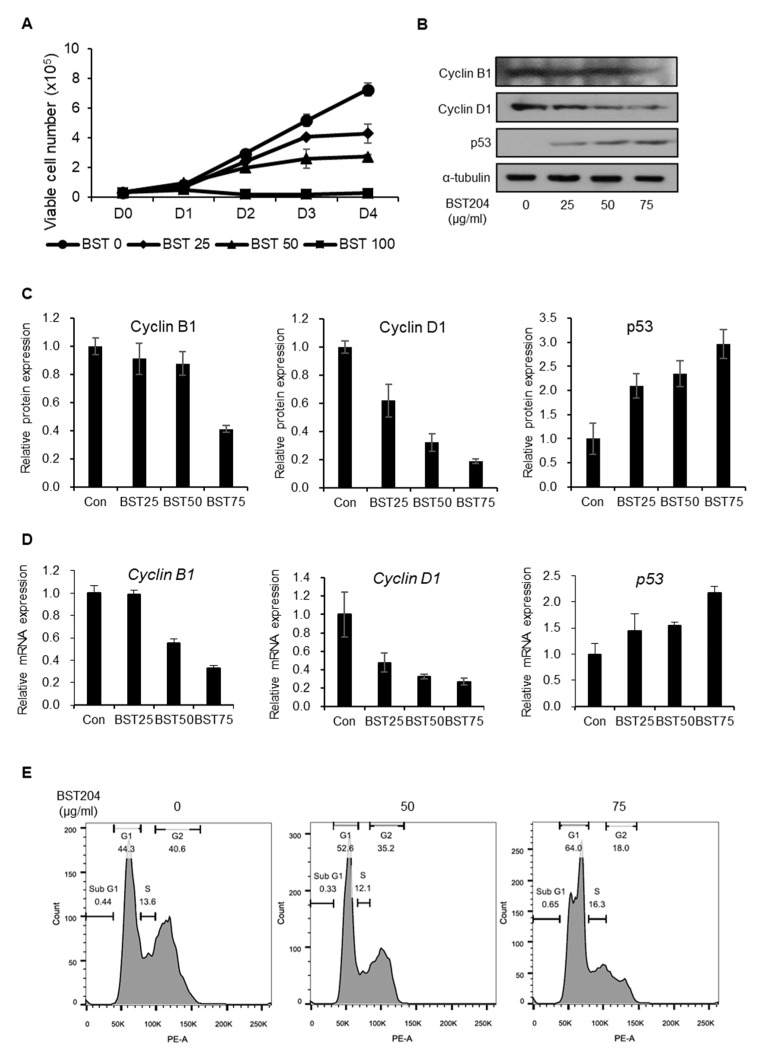
Effect of BST204 on embryonic carcinoma (EC) cell proliferation and cell cycle. (**A**) Proliferation assay of NCCIT cells. Cells were treated with the designated concentrations of BST204 for 4 days. The viable cell number was measured using a trypan blue exclusion assay. (**B**) Expression of cyclin B1, cyclin D1, and p53 was analyzed by immunoblotting. Alpha-tubulin was used as an internal control. (**C**) Relative expression of each protein was analyzed. (**D**) The mRNA levels of cyclin B1, cyclin D1, and p53 were analyzed by RT-qPCR. (**E**) NCCIT cells were treated with BST204, and cell cycle analysis was performed using flow cytometry.

In our previous study, we found that treatment with BST204 and its major ginsenoside component Rh2 leads to the inhibition of the proliferation and migration of colorectal carcinoma [29]. However, to the best of our knowledge, the effect of BST204 on CSC properties has never been investigated. Treatment with BST204 (25, 50, and 100 µg/mL) inhibited the proliferation of NCCIT cells in a dose-dependent manner (Figure 1A). NCCIT cells are testicular embryonic carcinoma with similar gene expression profiles to embryonic stem cells, with the abnormal overexpression of core stemness genes, including Nanog, Oct4, and Sox2. Upon treatment with BST204, the expression of the tumor suppressor protein, p53, was upregulated, while that of cyclin D1, a G1-dependent cell cycle protein, was downregulated, as determined by immunoblotting (Figure 1B,C). However, the expression of the G2-dependent cell cycle protein, cyclin B1, was marginally reduced upon treatment with 75 µg/mL BST204 and remained unaffected upon treatment with lower concentrations of BST204. These changes in the expressions of cell cycle proteins upon BST204 treatment correlated with their mRNA expression levels, indicating that BST204 affects the expression of cell cycle genes at the transcriptional level (Figure 1D). Due to these changes, EC cells were arrested at the G1 stage upon treatment with BST204 (Figure 1E). Dimethyl sulfoxide-treated NCCIT cells consisted of 44.3% G1 of the population, which was increased to 52.6% and 64% upon treatment with 50 µg/mL and 75 µg/mL BST204, respectively, explaining the suppression of EC proliferation through G1 cell cycle arrest.

### 2.2. BST204 Downregulates Cancer Stemness Protein Expression and Inhibits Tumorigenicity of EC Cells

**Figure 2:**
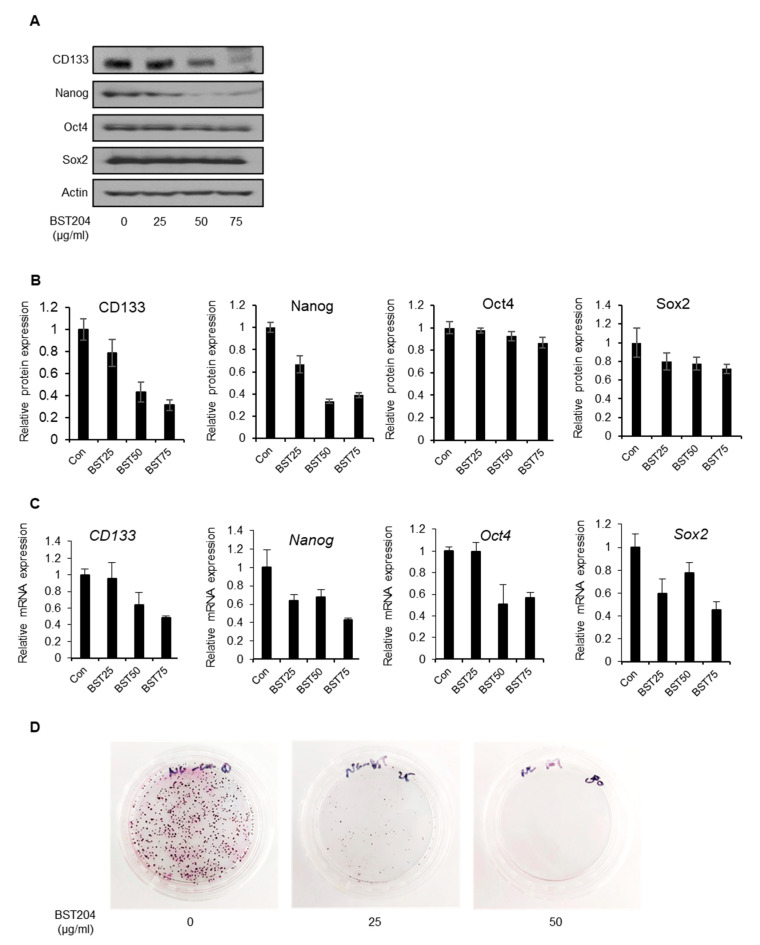
Effect of BST204 on cancer stemness gene expression and tumorigenesis. (**A**) Expression of CD133, Nanog, Oct4, and Sox2 was analyzed by immunoblotting. Actin was used as an internal control. (**B**) Relative expression of each protein was analyzed. (**C**) The mRNA levels of CD133, Nanog, Oct4, and Sox2 were analyzed by RT-qPCR. (**D**) In the colony formation assay, NCCIT cells were seeded in plates and incubated with BST204 until the formation of colonies. The colonies were stained with 0.5% crystal violet.

We have previously reported that the upregulation of p53 due to either genotoxic stress or a p53-stabilizing molecule leads to the repression of cancer stemness through the downregulation of the CSC marker CD133 [20]. In NCCIT cells, p53 has a very low expression due to its p53 (mut/-) status, however, p53 was upregulated by genotoxic stress or ectopic expression and was able to downregulate CD133 expression [20]. Upon treatment with BST204, the expression level of p53 was upregulated (Figure 1A), which further resulted in the downregulation of CD133 and stemness transcription factor expression (Figure 2A,B). The mRNA expression levels of CD133, Nanog, and Oct4 were correlated with their protein expression upon treatment with BST204, suggesting that the suppression of cancer stemness genes are regulated at the transcriptional level (Figure 2C). Our earlier studies have shown that the downregulation of either CD133 or Nanog can inhibit tumorigenesis [20,21]. As the treatment of EC cells with BST204, even at the lowest concentration, results in the downregulation of CD133 and Nanog expression, it could strongly suppress colony formation (Figure 2D). These data indicate that BST204 inhibits not only cancer cell proliferation—in a manner similar to conventional cancer drugs—but also the CSC property and tumorigenesis.

### 2.3. CD133 Knockdown Leads to Suppression of Tumorigenesis but Not Cancer Cell Proliferation

**Figure 3:**
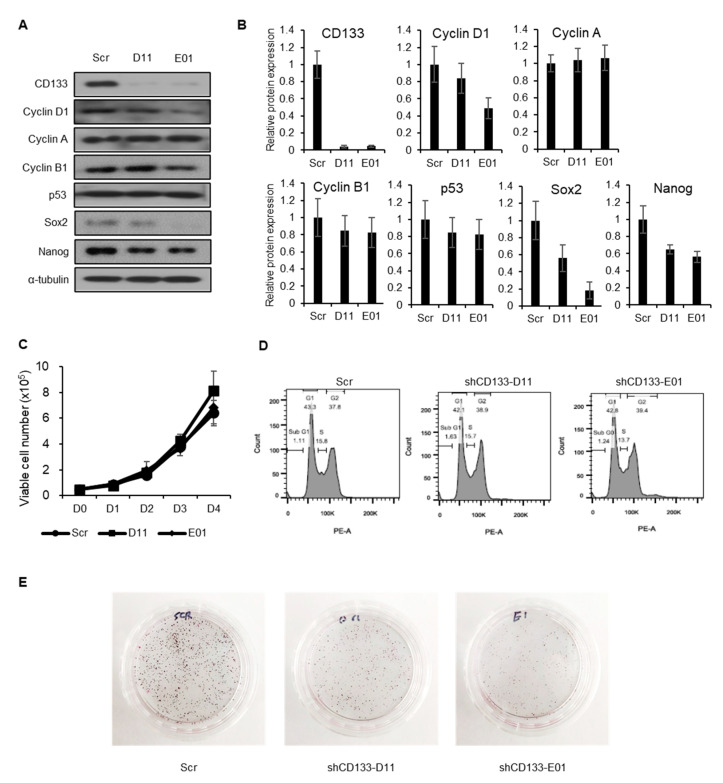
Effect of CD133 knockdown on embryonic carcinoma (EC) cell proliferation, cell cycle, and tumorigenesis. (**A**) Generation of stable CD133 knockdown cell lines was done using pLKO.1 (Scr) or shCD133-D11 (D11) or shCD133-E01 (E01). Expression levels of CD133, cyclin D1, cyclin A, cyclin B1, p53, Sox2, and Nanog were analyzed by immunoblotting. Alpha-tubulin was used as an internal control. (**B**) Relative expression of each protein was analyzed. (**C**) Proliferation assay of NCCIT control cells and NCCIT cells with CD133 knockdown. (**D**) Flow cytometry profiles of NCCIT control cells and NCCIT cells with CD133 knockdown. (**E**) For the colony formation assay, control NCCIT cells and NCCIT cells with CD133 knockdown were seeded in plates and incubated until colony formation. The colonies were stained with 0.5% crystal violet.

To determine whether the effects of BST204 on cancer proliferation and tumorigenesis are mediated through the downregulation of CD133, stable CD133-knockdown NCCIT cell lines were generated using two different short hairpin RNAs (shRNAs) against CD133. Both cell lines showed basal level expression of CD133 (Figure 3A,B). However, CD133 knockdown did not alter the expression of cell cycle-related proteins (p53, Cyclin D1, Cyclin A, Cyclin B1) that had been affected by BST204 treatment (Figure 3A,B). Accordingly, the knockdown of CD133 did not suppress cancer cell proliferation, or alter their cell cycle distribution (Figure 3C,D). Surprisingly, the knockdown of CD133 could suppress the colony forming ability of EC cells, suggesting that CD133 plays a pivotal role in tumorigenicity (Figure 3E). Thus, these results indicate that CD133 can serve as a target for drug development against tumorigenesis, which is the key property of CSCs.

### 2.4. BST204 Inhibits the Migration and Invasion of Cancer Cells via Suppression of Epithelial–mesenchymal Transition (EMT)

**Figure 4:**
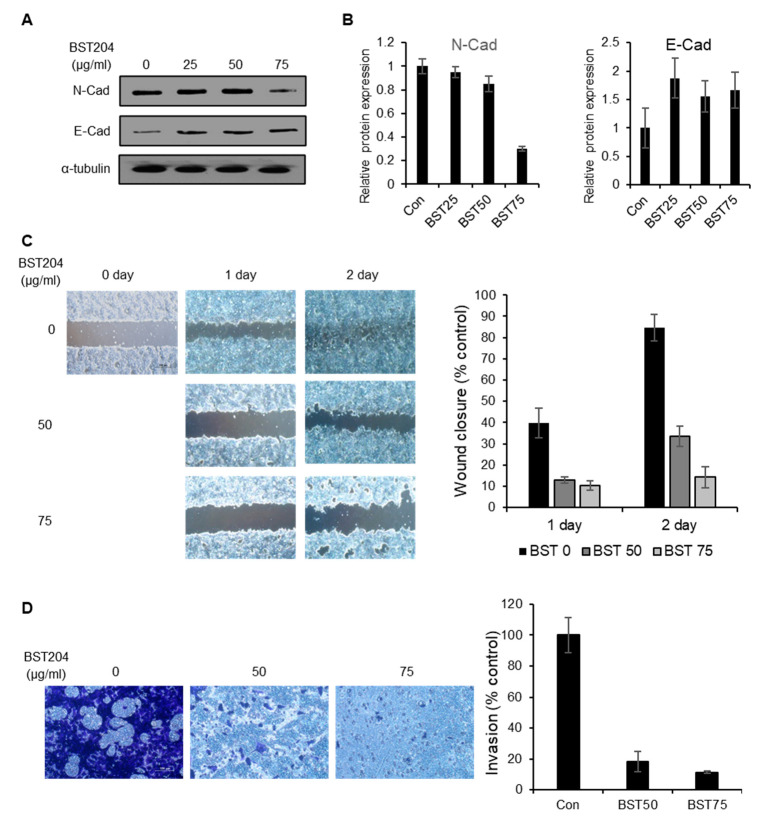
Effects of BST204 on EC cell migration and invasion. (**A**) Expression of N-cadherin and E-cadherin was analyzed by immunoblotting. Alpha-tubulin was used as an internal control. (**B**) Relative expression of each protein was analyzed. (**C**) The effects of BST204 on cell migration were evaluated by a wound healing assay. A wound was created by scratching the cell monolayer using a pipette tip. Cells were incubated with BST204 at the designated concentration for 2 days. Wound healing was captured using an Olympus IX70 microscope. (Scale bar: 100 µm) The quantification of wound closure (% control) is exhibited. (**D**) For the invasion assay, NCCIT cells were seeded (5 × 10^4^ cells/well) in a Matrigel-coated 24-well plate in the presence or absence of BST204 for 24 h. The invading cells were stained with 0.5% crystal violet and photographed using an Olympus IX20 microscope. (Scale bar: 100 µm). The quantification of the invasion (% control) is exhibited.

Tumor initiation or tumorigenicity is regarded as the key property of CSCs. Many studies have also suggested metastasis as another important characteristic of CSCs [32]. In fact, metastasis is the biggest cause of cancer-related deaths. Moreover, EMT is known to be more active in invasive or metastatic subsets of CSCs in many types of tumors [33,34]. First, we investigated the effect of BST204 treatment on the expression EMT-related proteins. Control (DMSO-treated) EC cells showed a high expression of N-cadherin, a mesenchymal marker, and a low expression of E-cadherin, an epithelial marker. Treatment with 75 µg/mL BST204 significantly reduced the expression of N-cadherin, whereas treatment with 25 µg/mL was sufficient to enhance the expression of E-cadherin (Figure 4A,B). Next, a wound healing assay and invasion assay were performed to determine whether the inhibition of EMT upon treatment with BST204 has a significant effect on EC cell migration and invasion. The migration of NCCIT cells was suppressed upon treatment with BST204 in a dose-dependent manner, and treatment with 75 µg/mL BST204 completely inhibited the migration of EC cells (Figure 4C). Furthermore, BST204 repressed the invasion of the EC cells in a dose-dependent manner, and this effect was apparent even at a concentration of 50 µg/mL (Figure 4D). These results suggest that BST204 inhibits the metastatic ability of CSCs through the suppression of EMT.

### 2.5. CD133 Knockdown Leads to Suppression of Migration and Invasion in EC Cells

**Figure 5:**
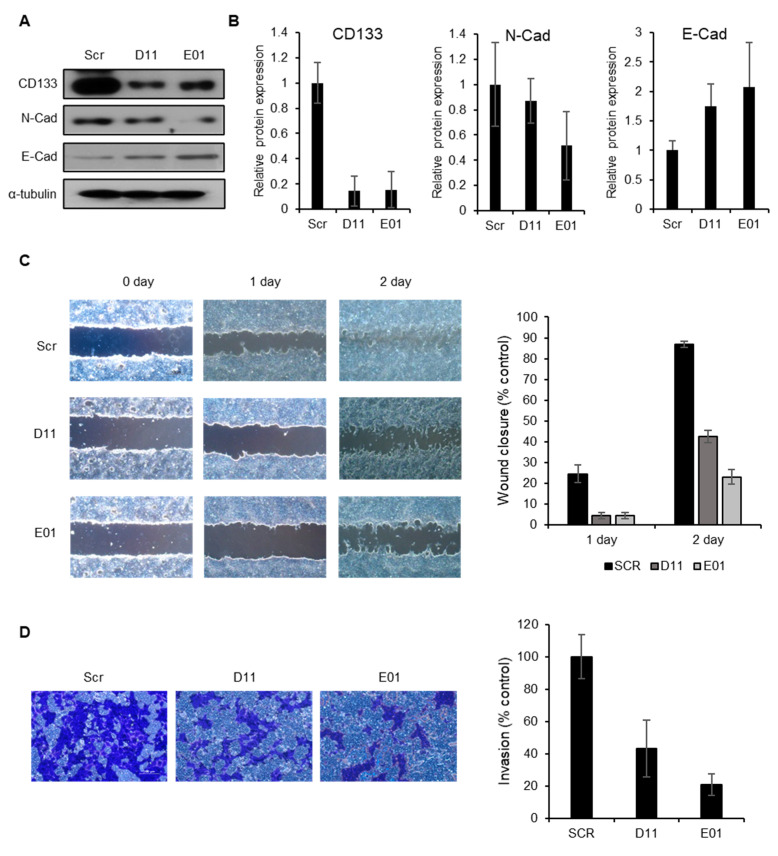
Effect of CD133 knockdown on EC cell migration and invasion. (**A**) Expression of CD133, N-cadherin, and E-cadherin was analyzed by immunoblotting. Alpha-tubulin was used as an internal control. (**B**) Relative expression of each protein was analyzed. (**C**) The effects of CD133 knockdown on cell migration were evaluated by a wound healing assay. A wound was created by scratching the cell monolayer using a pipette tip. Cells were incubated for 2 days. Wound healing was captured using an Olympus IX70 microscope. (Scale bar: 100 µm). The quantification of wound closure (% control) is exhibited. (**D**) For the invasion assay, control NCCIT cells and those with CD133 knockdown (5 × 10^4^ cells/well) were cultured in a Matrigel-coated 24-well plate for 24 h. Invading cells were stained with 0.5% crystal violet and photographed using an Olympus IX20 microscope. (Scale bar: 100 µm). The quantification of the invasion (% control) is exhibited.

CSCs expressing unique CSC markers, including CD133, have been identified as being responsible for the metastatic activities of CSCs [35,36]; however, the role of CSC markers in metastasis is not yet clearly understood. To determine whether the anti-metastatic effect of BST204 is mediated through the downregulation of CD133, we examined the expression of EMT markers upon CD133 knockdown. We found that the expression of N-cadherin was decreased, while that of E-cadherin was increased upon CD133 knockdown, indicating that CD133 can serve as a target for EMT suppression (Figure 5A,B). CD133 knockdown in cells—using shCD133-E01 shRNA—resulted in greater alterations in the expression of proteins involved in EMT than shCD133-D11. Similarly, both NCCIT cell lines with CD133 knockdown showed suppressed migration of EC cells, and the shCD133-E01 cell line with CD133 knockdown showed greater suppression of EC cell migration than the shCD133-D11 cell line (Figure 5C). The effect of CD133 knockdown on EC cell invasion also indicated that both CD133 knockdown cell lines have anti-invasive effects; however, the shCD133-E01 cell line has a stronger anti-invasive effect, probably due to a greater change in EMT protein expression in these cells (Figure 5D). These results support the hypothesis that the BST204-induced inhibition of metastatic ability could be mediated through the suppression of CD133.

### 2.6. Single Ginsenoside Components of BST204 have Limited Effects on Suppression of CSC Properties

**Figure 6:**
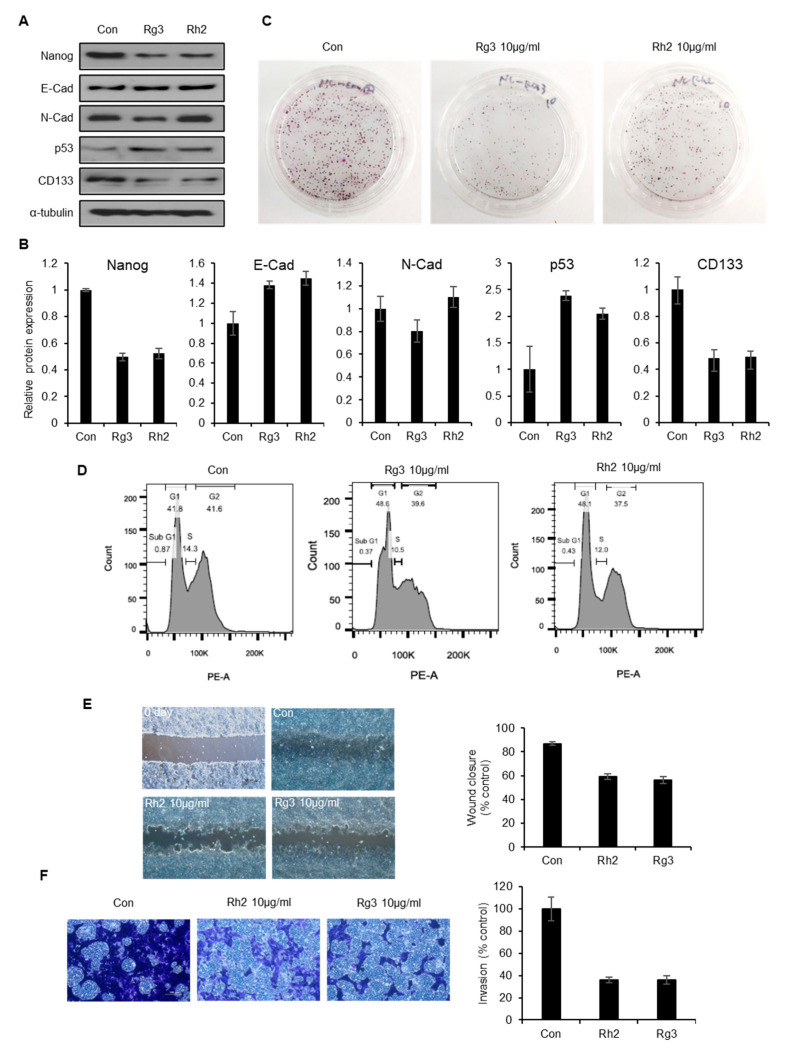
Effect of independent treatment with ginsenosides Rg3 and Rh2 on EC cell tumorigenesis, cell cycle, and migration. (**A**) Expression of Nanog, E-cadherin, N-cadherin, p53, and CD133 was analyzed by immunoblotting. Alpha-tubulin was used as an internal control. (**B**) Relative expression of each protein was analyzed. (**C**) For colony formation assay, NCCIT cells were seeded in plates and independently treated with Rg3 and Rh2 (10 µg/mL) and incubated until colony formation. The cells were then stained with 0.5% crystal violet. (**D)** NCCIT cells were treated with Rg3 and Rh2 (10 µg/mL) and cell cycle analysis was performed by flow cytometry. (**E**) The effects of Rh2 and Rg3 on cell migration were evaluated by the wound healing assay. A wound was created by scratching the cell monolayer with a pipette tip, before incubating the cells with Rh2 and Rg3 (10 µg /mL) for 2 days. Wound healing was captured using an Olympus IX70 microscope. (Scale bar: 100 µm). The quantification of wound closure (% control) is exhibited. (**F**) For the invasion assay, NCCIT cells (5 × 10^4^ cells/well) were seeded in a Matrigel-coated 24-well plate and incubated in the presence or absence of Rh2 or Rg3 for 24 h. Cells invading cells were stained with 0.5% crystal violet and photographed using an Olympus IX20 microscope. (Scale bar: 100 µm). The quantification of the invasion (% control) is exhibited.

Many studies have reported that single ginsenosides exhibit anticancer effects, including anti-tumorigenic, anti-proliferative, and anti-metastatic activities in a variety of tumors from different tissues [26,27,28]. As BST204 is a mixture of several ginsenosides, with Rg3 and Rh2 being the predominant active compounds, Rg3 and Rh2 comprise 10% and 5% of BST204 respectively [31]. As 75 µg/mL of BST204 treatment showed a significant inhibition of cell cycle, tumorigenesis and invasion were determined to investigate the effects of Rg3 or Rh2 at slightly higher concentrations than approximately proportional to that of BST204 (10 µg/mL, individually) on the expression of different proteins associated with cell cycle, stemness, and EMT (Figure 6A,B). Independent treatment with Rg3 and Rh2 increased the expression of p53; however, the increase was not as high as that induced by BST204. This trend held true for the changes in the expression patterns of CD133 and Nanog, whose expression decreased slightly in response to treatment with the single ginsenosides. The expression of E-cadherin was slightly increased upon treatment with Rg3 or Rh2, while that of N-cadherin was decreased only upon treatment with Rg3. Next, we determined the effects of single ginsenosides on tumorigenesis in NCCIT cells (Figure 6C). The anti-tumorigenic activity of Rg3 was moderate, while that of Rh2 was weak relative to that of Rg3 or BST204. Similarly, treatment with Rg3 resulted in slightly better G1 cell cycle arrest than that with Rh2, but it was less than that observed upon treatment with BST204 (Figure 6D). Treatment with Rg3 and Rh2 at the concentration of 10 µg/mL increased the G1 population from 41.8% to about 48%, while the treatment of NCCIT cells with BST204 at a concentration of 75 µg/mL resulted in an increase in the G1 population to 64%. Compared to BST204, single ginsenosides could show only modest effects with respect to migration and invasion as well (Figure 6E,F). Taken together, though the single ginsenosides Rg3 and Rh2 exert anti-CSC effects, BST204 seems to have a greater synergistic effect against CSCs.

## 3. Discussion

*Panax ginseng* (Asian ginseng), *P. quinquefolius* (American ginseng), and *P. notoginseng* (notoginseng) are the three ginseng families that are commonly used as a health supplement or herbal medicine. All three species contain a unique set of pharmacological compounds known as ginsenosides, which are classified into two structural groups: panaxadiols (Rb1, Rb2, Rc, Rd, Rg3, Rh2, and Rh3) and panaxatriols (Re, Rf, Rg1, Rg2, and Rh1), and are extensively studied for their pharmacological effects [37]. The quality and composition of ginsenosides among these families, as well as the process of their isolation and purification, differ widely. For instance, red ginseng is processed by heating or steaming, which enriches Rg3 levels, and improves its anticancer effects [38]. Similarly, fermentation with β-glucosidase increases the levels of minor ginsenosides, including Rg3, Rh2, and F2, which show better pharmacological activities than the major ginsenosides in the raw state [39,40,41,42]. In this study, we examined the effects of a fermented ginseng extract, BST204—that has higher Rh2 and Rg3 contents—on the properties of CSCs. Many studies on the pharmacology of ginseng products, including BST204, have focused on conventional anticancer activities, such as the induction of apoptosis and cell cycle arrest [24,26,28,43]. However, the effect of ginseng products or ginsenosides on CSC properties has not been explored much yet. Here, we demonstrated that the treatment of EC cells with BST204 leads to G1 cell cycle arrest, as well as the inhibition of tumorigenesis (Figure 1). Cell cycle arrest and the suppression of cell proliferation in the background of BST204 treatment were mediated through the induction of p53, which is a well-established mechanism in many cancer drugs. Interestingly, the anti-tumorigenic property seems to be mediated through the inhibition of the CSC marker CD133, as demonstrated in CD133-knockdown NCCIT cell lines (Figure 3).

CSCs are defined as a small population of tumor cells, which have self-renewal capacity and can give rise to a heterogeneous subpopulation of cancer cells that comprise the tumor. Due to their ability of propagating tumor cells, they are often referred to as “tumor-initiating cells” and tumorigenicity is their main feature. Unlike the fast-growing major population of the cancer cells, CSCs are maintained in a quiescent, slow-growing state, which is the mechanism through which they can escape and survive the conventional anti-proliferative therapy or radiotherapy, which target the rapidly proliferating cells. Because of these characteristics, they can cause cancer relapse and drug resistance. Therefore, targeting CSCs is the next avenue to explore when thinking about eradicating tumors. Many CSC markers have been identified and several signaling pathways, including neurogenic locus notch homolog protein (Notch), sonic hedgehog (SHH), and Wnt/β-catenin, are required by CSCs to maintain their stemness [44,45]. These signaling pathways have been validated as CSC drug targets, and many small molecules are under clinical trials. Recently, targeting cancer stemness was shown to suppress cancer relapse and metastasis [46,47]; however, there is no established protocol for targeting CSC markers as of yet. Here, using BST204, we validated CD133 as a possible CSC target.

Metastasis refers to the spread of cancer cells from the site of the primary tumor to other sites in the body and it is known to be a major obstacle in combating cancer. It increases the morbidity and mortality of patients with cancer and accounts for 90% of cancer deaths. EMT is a transdifferentiation program in which epithelial cells—the surface barrier—transform into mesenchymal cells—scaffolding or anchoring cells—that play a role in tissue repair and wound healing. Accumulating evidence suggests that EMT is aberrantly activated in CSCs, and that there is a correlation between the high expression of stemness-related transcription factors or CSC markers and EMT proteins [48,49]. For example, CD133 expression can induce EMT and increase metastasis in pancreatic cancer, and CD133^+^CXCR^+^ CSCs have been proven to be essential for pancreatic cancer metastasis [35,50]. However, a strategy for targeting CSC markers involved in EMT and the suppression of metastasis has not been well established. In our study, we show that treatment with BST204 significantly inhibits EMT and EC invasion through the downregulation of N-cadherin, a mesenchymal cell marker, and the upregulation of E-cadherin, an epithelial cell marker. By knocking down CD133 in NCCIT cell lines, we identified CD133 to be involved—at least partially—in the regulation of EMT, cancer migration, and invasion.

Recently, a few studies have reported that the ginsenosides Rh2 and Rg3 could inhibit CSC properties in skin squamous cell carcinoma and breast cancer through reducing the number of Lgr5-positive cells or regulating self-renewal activity, respectively [51,52]. Ginsenosides Rh2 and Rg3 have also been reported to have an effect on the immune system, which can attenuate cancer resistance or improve cancer drug-induced immune competence [53,54]. We treated EC cells with Rh2 and Rg3 and found that treatment with either compound moderately inhibited CSC properties such as tumorigenesis, EC migration, and invasion (Figure 6). It is possible that minor ginsenoside compounds also contribute to the regulation of CSC markers and the combined treatment of ginsenosides may show greater synergistic effects on the suppression of CSC characteristics. In fact, it was recently reported that the combined treatment of protopanaxatiol and the ginsenoside Rh2 shows a synergistic effect on antiproliferative activity in breast cancer cells [55]. We further showed that the inhibitory effect of BST204 on EC cells is exerted through the suppression of CD133 expression, and this leads to the abrogation of both tumorigenesis and tumor invasion. Difficulties in the chemical synthesis of Rh2 or Rg3, and more importantly the synergistic anti-CSC effects exerted by BST204 in an unpurified form, make it a more effective therapeutic option for the treatment of cancer.

## 4. Materials and Methods

### 4.1. Preparation of BST204

BST204 was provided by Green Cross Well Being Co. Ltd., (Seongnam, Korea), and was manufactured as described in an earlier study [32]. Briefly, the harvested ginseng was extracted with ethanol followed by incubation with ginsenoside-β-glucosidase. The reactant was purified using HP20 resin (Mitsubishi Chemical, Tokyo, Japan), followed by washing with distilled water and 95% ethanol. The ethanol fractions were concentrated and were designated as BST204. The composition of the ginsenosides in BST204 has been specified in an earlier study [31].

### 4.2. Cell Culture and Proliferation Assay

NCCIT cells (American Type Culture Collection, Manassas, VA, USA) were cultured in RPMI 1640 (Welgene Inc., Gyeongsan-si, Korea), supplemented with 10% fetal bovine serum (FBS; Welgene Inc., Gyeongsan-si, Korea) and 1% penicillin/streptomycin (Welgene Inc., Gyeongsan-si, Korea). Cells (5 × 10^5^) were seeded in a six-well plate and cultured in the presence of BST204 (25, 50, or 100 µg/mL) or DMSO (0.1% as control) for 4 days. Cells were detached using trypsin-EDTA, washed with ice-cold phosphate-buffered saline, and stained with 0.4% trypan blue solution (Thermo Fisher, Waltham, MA, USA). Live cells were counted using a hemocytometer with three independent biological replicates, and the counts are presented as mean ± SEM.

### 4.3. Immunoblotting

Cells were grown in 10 cm dishes, and upon reaching a confluency of 80%, were treated with designated compounds (0.1% DMSO, BST204, Rh2, Rg3), incubated for 24 h, and lysed in 150 µL of Proprep (iNtRON, Seongnam-si, Korea). The lysate was centrifuged at 12,000× *g* for 10 min at 4 °C. Samples from each supernatant were treated with 4× Laemmli buffer (Bio-Rad, Hercules, CA, USA), separated by SDS–polyacrylamide gel electrophoresis, and transferred onto a polyvinylidene difluoride membrane (Merck Millipore, Burlington, MA, USA). These membranes were incubated overnight at 4 °C with the relevant primary antibodies (see Section 4.4, Reagents and Antibodies), followed by incubation with horseradish peroxidase-conjugated secondary antibodies for 1 h (Abcam, Cambridge, UK). The chemiluminescent signals were detected using an enhanced chemiluminescence system (iNtRON, Seongnam-si, Korea). Densitometry from three different immunoblots was measured using ImageJ software, normalized relative to actin expression and is presented as means ± SEM. The raw data of western blots are provided in Figure S1.

### 4.4. Reagents and Antibodies

Anti-p53 clone DO-1, anti-E-cadherin, anti-N-cadherin (Santa Cruz Biotechnology, Dallas, TX, USA), anti-PROM1 (CD133) (Abnova, Taipei, Taiwan), anti-Nanog, anti-Oct4, anti-Sox2 (Abcam, Cambridge, UK), and anti-actin (Merck Millipore, Burlington, MA, USA) antibodies were used as primary antibodies for immunoblotting. For treatments, 20(S)-ginsenoside Rh2 (> 98%) and 20(S)-ginsenoside Rg3 (> 98%) (Abcam, Cambridge, UK) were used.

### 4.5. Wound Healing Assay

NCCIT cells were seeded in a six-well plate and grown to confluency. The cells were scratched with a sterile pipette tip to create a wound, and were then treated with BST204 (50, 75 µg/mL), Rh2 (10 µg/mL), or Rg3 (10 µg/mL) and photographed after 1 or 2 days using a microscope (Olympus IX70, Olympus, Tokyo, Japan). The distance between the wound from three different fields was measured and analyzed using ImageJ software, and the ratio was normalized relative to time point 0 days.

### 4.6. Matrigel Invasion Assay

The invasion capacity of tumor cells was assessed using Transwell chambers (Corning Costar, Corning, NY, USA) containing 6.5 mm polycarbonate filters (8 μm pore size) according to the manufacturer’s instructions, except that each filter was coated with 100 μL of Matrigel (1:20 dilution in cold Dulbecco’s modified Eagle’s medium) to form a thin continuous film on the top of the filter. NCCIT cells (100 μL containing 5 × 10^4^ cells) in DMEM containing 10% FBS were transferred to each well. After incubating with BST204, Rg3, Rh2, or DMSO (0.1% as control) for 24 h at required concentrations, the cells were stained and imaged using a microscope (Olympus IX70, Olympus, Tokyo, Japan). The stained cells were then quantified from three different fields using ImageJ software, and the ratio was normalized relative to the control cells or scramble plasmid-transfected cells.

### 4.7. Clonogenic Colony Formation Assay

To examine the tumorigenicity of undifferentiated CSC-like cells, a clonogenic assay was performed. Cell suspensions were diluted in DMEM supplemented with 10% FBS, and 1000 cells/well were plated in six-well plates. Cells were treated with BST204 (50, 75 µg/mL), Rh2 (10 µg/mL), or Rg3 (10 µg/mL) and allowed to grow for 8 d to form colonies. Then, the cells were fixed using 4% paraformaldehyde (Electron Microscopy Science, Hatfield, PA, USA) and stained with 0.5% crystal violet (Sigma-Aldrich, St. Louis, MO, USA). Each plate was photographed using an Olympus IX70 microscope.

### 4.8. Flow Cytometry

Cell cycle distribution upon treatment with BST204, Rg3, Rh2, or upon CD133 knockdown was determined by flow cytometry after staining the cells with propidium iodide (Sigma-Aldrich, St. Louis, MO, USA). NCCIT cells (5 × 10^5^) were seeded and treated with BST204 (50, 75 µg/mL), Rh2 (10 µg/mL), Rg3 (10 µg/mL), or DMSO (0.1% as control) for 24 h. After incubation, both floating and attached cells were collected. Then, the cells were stained with propidium iodide, and cell cycle distribution was determined using a BD Canto II flow cytometer and analyzed using FlowJo software (Tree Star software, San Carlos, CA, USA).

### 4.9. Generation of Stable CD133 Knockdown Cell Line

The generation of stable CD133 knockdown cell lines has been described in an earlier report [20]. Briefly, a pLKO.1 lentiviral vector containing CD133 shRNA was purchased from Open Biosystems. Empty pLKO.1 (Scr) or shCD133-D11 (D11) or shCD133-E01 (E01) pLKO.1 were transfected into HEK293T cells, the supernatant containing the virus was transfected into the NCCIT cells, and stably infected cells were selected using 10 µg/mL puromycin (Sigma-Aldrich, St. Louis, MO, USA).

### 4.10. Quantitative Reverse Transcription Polymerase Chain Reaction (RT-qPCR)

RNA extracts were prepared as previously described using Easy-Blue reagent (iNtRON, Seongnam-si, Korea). Then, 1 μg of total RNA was reverse transcribed into cDNA using a reverse transcription kit (Promega, Madison, WI, USA). Quantitative real-time PCR was performed using KAPATM SYBR FAST qPCR (KAPA BIOSYSTEMS, Wilmington, MA, USA) with the CFX96™ or Chromo4™ real-time PCR detector (Bio-Rad, Hercules, CA, USA). The relative mRNA levels were normalized to the GAPDH mRNA levels for each reaction. The sequences of the primers used are as follows: GAPDH forward, 5′-GAGTCAACGGATTTGGTCGT-3′; GAPDH reverse, 5′-TTGATTTTGGAGGGATCTCG-3′; cyclin D1 forward, 5′-GAGAAGTTGTGCATCTACACTG-3′; cyclin D1 reverse 5′-AAATGAACTTCACATCTGTGGC-3′; cyclin B1 forward, 5′-AAGAGCTTTAAACTTTGGTCTGGG-3′, cyclin B1 reverse, 5′-CTTTGTAAGTCCTTGATTTACCATG-3′; p53 forward, 5′-TGCGTGTGGAGTATTTGGATG-3′, p53 reverse, 5′-TGGTAAGTCAGCCAACCTC-3′; CD133 forward, 5′-CAGAGTACAACGCCAAACCA-3′, CD133 reverse 5′-AAATCACGATGAGGGTCAGC-3′; Oct4 forward, 5′-GAGATATGCAAATCGGAGACC-3′, Oct4 reverse, 5′-GCCTGGAGCACCAAAGTG-3′; Sox2 forward, 5′-CACAACTCGGAGATCAGCAA-3′, Sox2 reverse, 5′-CTCCGGGAAGCGTGTACTTA-3′; Nanog forward, 5′-GCCCTGAGAAGAAAGAAGAG-3′, Nanog reverse, 5′-CGTACTGCCCCATACTGGAA-3′; E-cadherin forward, 5′-GGTTATTCCTCCCATCAGCT-3′, E-cadherin reverse, 5′-CTTGGCTGAGGATGGTGTA-3′; N-cadherin forward, 5′-GCGTCTGTAGAGGCTTCTGG-3′, N-cadherin reverse, 5′-GCCACTTGCCACTTTTCCTG-3′.

## 5. Conclusions

Treatment with BST204 leads to the upregulation of the tumor suppressor protein, p53, which promotes G1-S cell cycle arrest and inhibits cell proliferation in a manner similar to that observed upon using conventional cancer drugs. Importantly, the induction of p53 by this treatment leads to the downregulation of the CSC marker CD133, the expression of which is correlated with that of core stemness-related transcription factors and regulatory proteins involved in EMT, thereby leading to the suppression of tumorigenesis and metastasis

## Supplementary Materials

The following are available online, Figure S1: Raw data of Western blots.
