# Supplementary material for: Fermented Ginseng Extract, BST204, Suppresses Tumorigenesis and Migration of Embryonic Carcinoma through Inhibition of Cancer Stem Cell Properties
Source: Molecules. 2020 Jul 8;25(14):3128. doi: 10.3390/molecules25143128 (PMC7397298; doi:10.3390/molecules25143128)

Supplementary Figure 1 Raw data of Western blots. (A) Raw data of western blots used in Figure 1B. (B) Raw data of western blots used in Figure 2A. (C) Raw data of western blots used in Figure 3A. (D) Raw data of western blots used in Figure 4A. (E) Raw data of western blots used in Figure 5A. (F) Raw data of western blots used in Figure 6A.

A

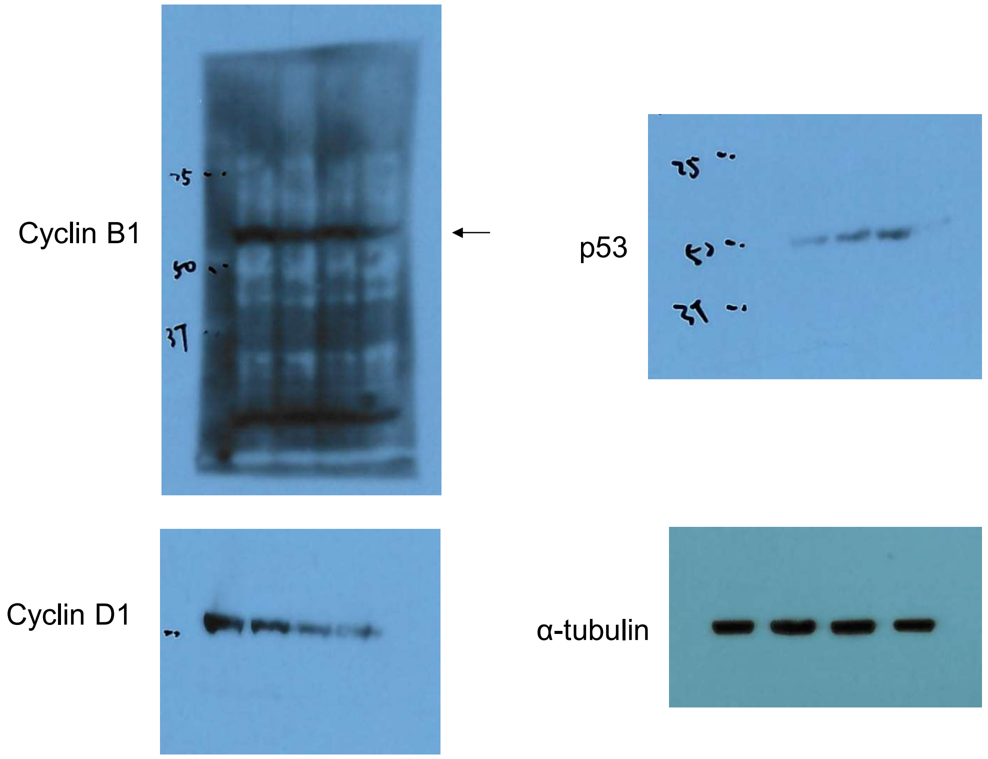

B

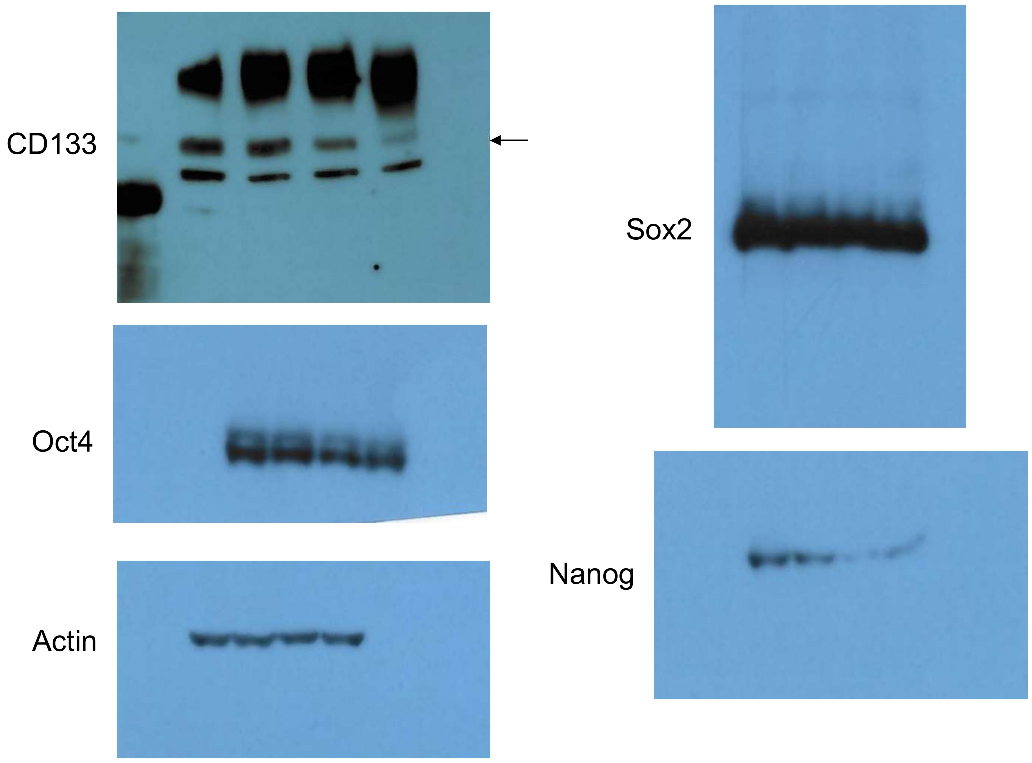

Supplementary Figure 1 continued

C

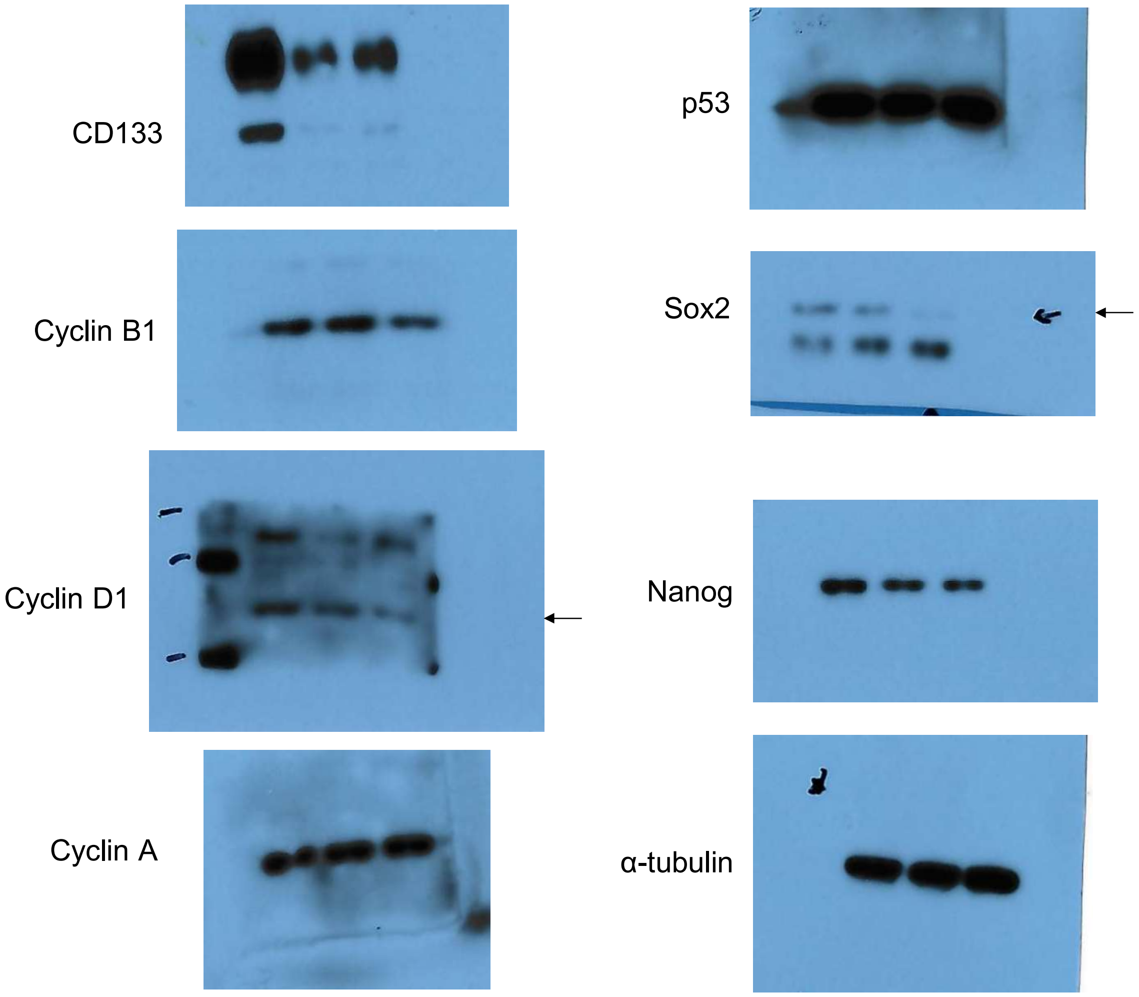

D

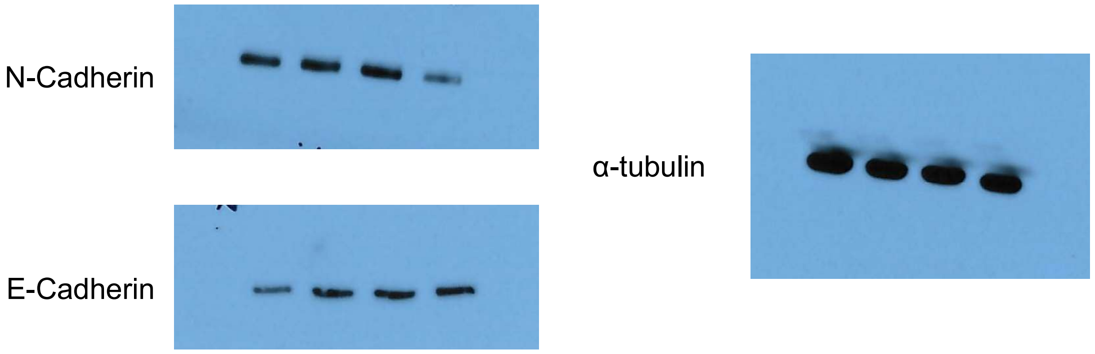

Supplementary Figure 1 continued

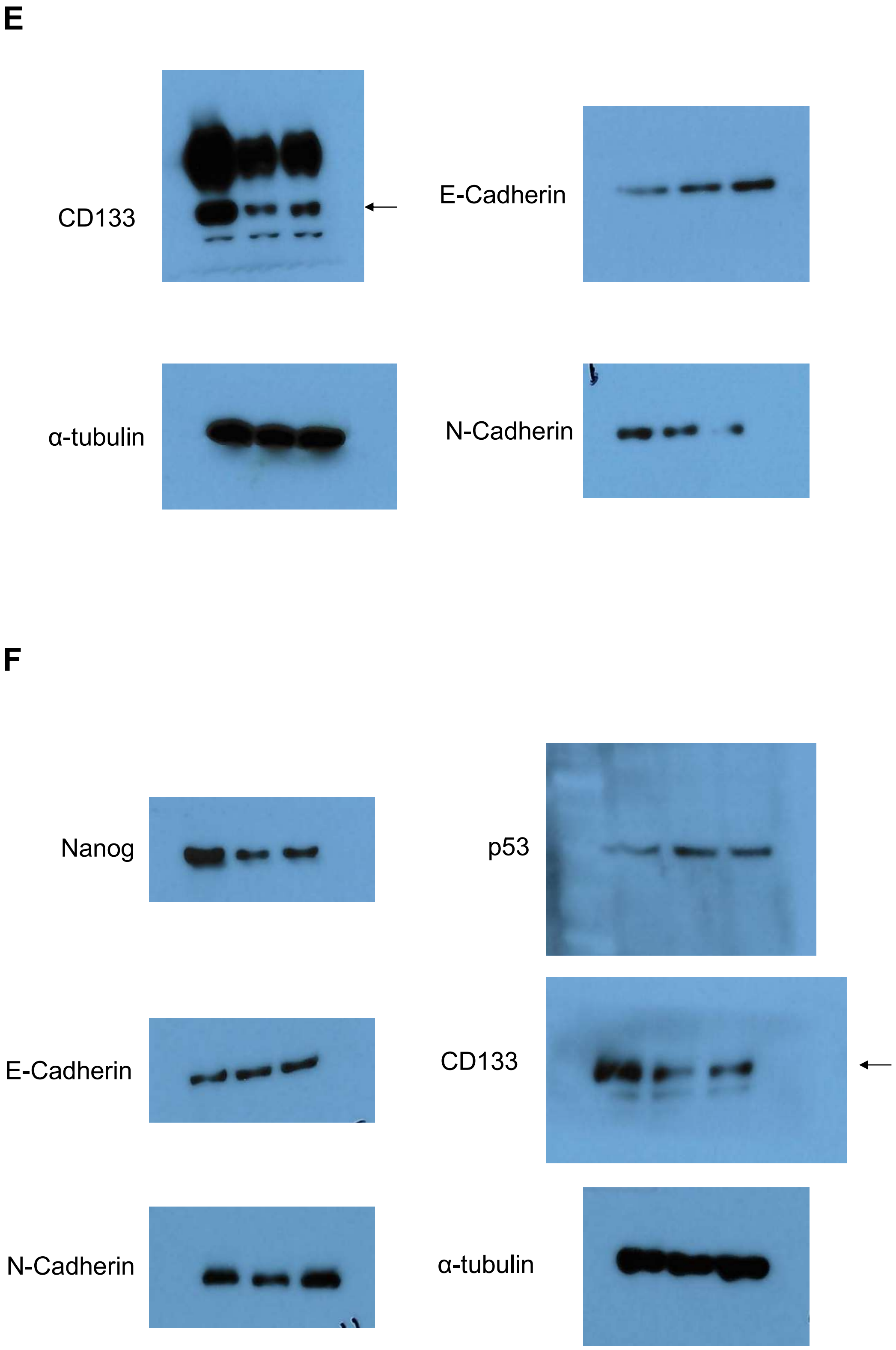

Supplement: Supplementary file 1 [file molecules-25-03128-s001.pdf]
